# Benefits of Incobotulinumtoxin A in Pain, Spasticity and Functionality—A 5 Years Review

**DOI:** 10.3390/toxins18070291

**Published:** 2026-07-03

**Authors:** André Cruz

**Affiliations:** 1 Department of Physical and Rehabilitation Medicine, Unidade Local de Saúde do Alto Minho, Estrada deSanta Luzia, 4904-858 Viana do Castelo, Portugal; andre.cruz@ulsam.min-saude.pt; 2 School of Medicine, University of Minho, 4710-057 Braga, Portugal

**Keywords:** spasticity, pain, botulinum toxin, rehabilitation

## Abstract

Introduction: Pain is a major contributor to functional impairment in neurological patients. Botulinum toxin type A (BT-A) is well-established for focal spasticity and has shown some benefits in pain reduction. Long-term data from large cohorts remain limited. Material and Methods: This retrospective study (2017–2022) analyzed 20 variables from spastic patients treated with Incobotulinumtoxin A (INCO) for pain and spasticity. Functional analysis was classified into 5 major domains: Pain, Spasticity spectrum, Mobility, Activities of daily living, and Gait. Results: A total of 1575 records from 67 patients were collected. The mean age was 57.7 years, mostly female (*n* = 35), and stroke was the leading etiology (*n* = 40). The most typical patterns were wrist flexion (80%) and ankle flexion (94.9%). It was injected with 50 to 400 units of INCO, targeting an average of 3.47 upper-limb and 2.34 lower-limb muscles per session. Functional improvement was observed across all domains (GAS change 27.4), with the greatest effects in pain (GAS change 26.9; 5.16 VAS reduction). Discussion: The findings, derived from one of the largest datasets, support that INCO, and probably BT-A, should be indicated for the pain of spastic patients, regardless of etiology. Conclusions: Promising functionality outcomes, especially in this group, warrant further, more comprehensive studies.

## 1. Introduction

Spasticity and pain are two frequently found problems in neurological diseases that can interfere with patients’ quality of life [1] and rehabilitation process [2].

Spasticity is a velocity-dependent increase in muscle tone or tonic stretch reflexes associated with hypertonia [3], and its prevalence varies depending on the underlying condition, ranging from 2–350 per 100,000 in multiple sclerosis to 40–600 per 100,000 in stroke [4]. The presentation of spasticity is variable and may slightly interfere with movement or, in extreme cases, produce an immobilized articulation. Besides, spastic patients also experience hyperkinetic movements, such as spasms, clonus, focal dystonia, and myoclonus [5,6], and these hyperactive movements can cause discomfort and interfere with patients’ quality of life [5,6].

Pain is a very prevalent complaint, especially in spastic patients, with a prevalence up to 70% in SCI and stroke [5,7] and can be linked to different etiologies (musculoskeletal, central pain and spasticity-related). The pathophysiology of pain in patients with spasticity is likely multifactorial, involving muscle overactivity, ischemia, contractures, osteoarticular deformities, soft-tissue inflammation, and both central and peripheral sensitization mechanisms [8].

Pain and spasticity engender patients’ higher levels of depression and worse quality of life [7,9]. Taking into consideration these facts and the high prevalence of these symptoms, control of the spasticity spectrum (spasticity and hyperkinetic movements) and pain is vital for improving the quality of life of our patients.

Many treatments have been developed to treat pain and the spasticity spectrum. In the field of rehabilitation, conservative measures, such as massage, stretching, and reeducation of movement techniques, are the mainstream of treatment. Splints can help in some cases. Thermotherapy and electrotherapy are also commonly used [10,11]. In this field, particularly extracorporeal shockwaves seem to have a promising impact on spasticity control, increasing efficacy and prolonging the effects of botulinum toxin applications [12,13]. Oral treatments like analgesics and antispastics (e.g., baclofen and tizanidine) are widely used but cannot treat a specific location, need higher doses with more side effects, and are potentiated by multiple interactions with other necessary treatments for the primary neurological disease [14].

Botulinum toxin type A have been reported to have great benefits in focal spastic patients, with level A recommendation in these conditions [15], but the benefit in pain in these patients is still scarce, reported in small samples and mainly in less than a year of follow-up.

The lack of perfect control over these symptoms leaves people suffering.

Difficulties in treatment result from many variables: different types of pain, associated motor and sensory impairments, different tonus alterations, and the perception of pain itself, which varies from person to person. In fact, pain is a difficult symptom to analyze and to establish the best treatment because it is something we cannot see. Merskey, the chair of the IASP Subcommittee on Taxonomy, defines it as “a psychological concept and not a physical measure” [16,17], and despite the actual IASP definition of pain, it is still very dependent on what the patient reports and is not an exact measure.

For all these facts, pain and spasticity can reduce rehabilitation outcomes and recovery, and so produce an impact on the restoration of function.

According to a PubMed analysis, including the terms “Pain,” “Spasticity,” and “Botulinum toxin,” there were 103 studies of revision or randomized controlled trials in the last ten years, of which 14 studies directly analyzed the treatment of pain in spasticity patients with botulinum toxin. Sample sizes were small (the majority < 40 patients), follow-up periods (the majority up to 12 weeks), and different injection sites/dosages/measures were found [18,19,20,21,22,23,24,25,26,27,28,29,30]. Meanwhile, the majority of studies show a positive impact of botulinum toxin on spastic patients’ pain from different etiologies [28,29,30,31,32].

The benefits of botulinum toxin in pain seem to result from the reduction of pain mediators, such as substance P, calcitonin gene-related peptide (CGRP), somatostatin, serotonin, and bradicinine [33,34] in the area where it is applied but also from distant action at the central nervous system [35], modulating peripheral and central processing pathways.

The objective of this study was to analyze the benefits of Incobotulinumtoxin A (Xeomin^®^, Merz Pharmaceuticals GmbH, Frankfurt, Germany) on pain and function in spastic patients in a larger spasticity and pain sample, with a long follow-up period, and to evaluate variables, with special focus on pain, that may influence the results and their possible relationships.

## 2. Results

A total of 67 patients were included in the study. From these patients, we collected 1575 records that were transferred to Excel spreadsheets; many of them contained multiple variables and scores.

The mean age was 57.7 ± 13.6 years, and the most common gender was female (n = 35, 52.2%). The most common cause of treatment was stroke (n = 40, 59.7%), followed by cerebral palsy and spinal cord injury (n = 9, 13.4%) (Table 1).

In each appointment, a mean of 3.47 ± 1.34 muscles was treated in the upper limb and 2.34 ± 1.09 in the lower limb. The usual pattern of the upper limb was wrist (80%) and finger flexion (75.6%), while in the lower limb it was ankle flexion (94.9%) and knee extension (44.1%) (Table 2).

The lowest dose used was 50U, and the maximum was 400U. The mean of the minimum dose in the sample was 126.3 ± 52.8, and the mean of the maximum dose was 208.6 ± 73.6. The number of muscles treated in each session ranged from 1 to 10 (mean minimum of 2.91 ± 1.33; mean maximum of 5.70 ± 2.09), and according to the evaluation, different muscles were selected during the total follow-up period, for a total of 14 different muscles (total of muscles in all sessions mean 6.21 ± 2.60).

Of these 67 spastic patients, pain was present at the beginning and during the follow-up in most of the patients—37 (55.2%)—of those, mainly were stroke related (64.9%). The other etiologies of spastic patients that presented pain were cerebral palsy (13.5%), spinal cord injury (13.5%), multiple sclerosis (2.7%), traumatic brain injury (2.7%), or other causes (2.7%).

Mean pain levels were high (7.1 ± 1.8). Moderate pain was found in 48.6% of the population and severe pain in 51.4%. There was a 5.16 ± 2.4 points median reduction from baseline in pain score in general and in the different populations at the one-month reevaluation appointment, and similar results were found and maintained during the 5-year course of this study, each time after new treatments.

**Figure 1:**
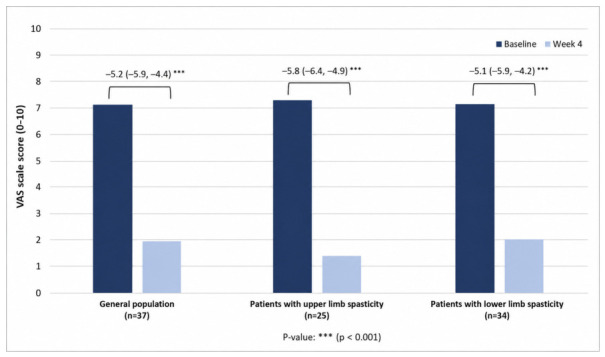
VAS scale score changes according to upper and lower limb spasticity.

For upper and lower limbs, change was −5.84 ± 2.2 and −5.09 ± 2.4, respectively (Figure 1).

**Figure 2:**
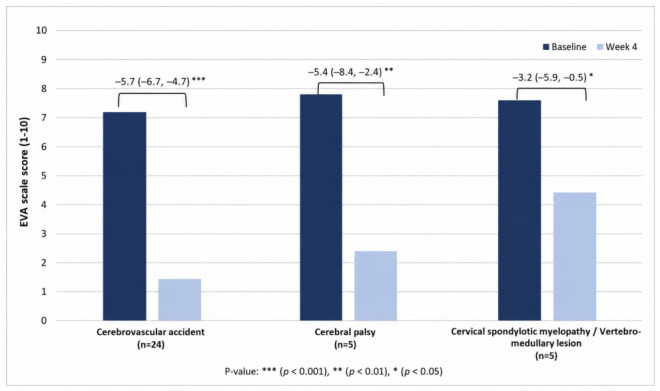
VAS scale score changes in different etiologies.

Pain reduction was found in all etiologies: stroke (−5.71 ± 2.3), cerebral palsy (−5.40 ± 2.4), and spinal cord injury lesion (−3.20 ± 2.2) (Figure 2).

Overall, all patients had benefits across the 5 categories, but the greatest impact was found in pain, followed by gait and mobility.

The majority of patients improved in at least one category (92.5%), and most achieved the objectives (97.2% for pain, 94.5% for gait, 93.1% for mobility, 81.3% for ALDs, and 90.9% for spasticity spectrum) (Figure 1).

Accumulated mean GAS T-score was 63.8 ± 7.7. When analyzing the 5 studied fields, we found GAS T-scores of 66.9 (±6.2) in pain, 62.8 (±8.1) in gait, 57.8 (5.6) in mobility, 54.1 (±8.0) in ALDs, and 58.2 (±6.0) in spasticity spectrum (Figure 2).

Overall, the GAS change was 27.4 (±7.9) points higher than basal. GAS pain-related score improvement was 26.9 (±6.2), and gait was 22.8 (±8.1). GAS change related to mobility, ALDs, and the spasticity spectrum was less intense, with values of 17.76 (±5.63), 14.06 (±7.98), and 18.18 (±6.03), respectively (Figure 1).

Most patients improve much more than expected (+2) in pain (77.1%) and gait (47.1%), and slightly more than expected in spasticity spectrum (90.9%), mobility (90.7%), and ALDs (73.1%).

Slight pain was common during application, but we did not observe major side effects during or after the procedures in this large number of applications.

After a univariate analysis, a statistically significant correlation was found between pain scores and the presence of spasticity in the upper limbs (*p* = 0.009) and the number of superior limbs affected (*p* = 0.022), but this data was not confirmed by multivariate analysis. All the other factors analyzed have not shown correlation to changes in pain, spasticity spectrum, mobility, activities of daily living, and gait.

## 3. Discussion

According to the present study, the benefits of INCO were maintained during a long period (5 years) and across multiple functional domains related to function and pain. The sustained and successful therapeutic effect throughout the 5-year follow-up period may be attributed to several factors, including appropriate dosing, optimal muscle targeting, precise injection technique, adequate treatment intervals, careful patient selection, appropriate clinical indication, and low immunogenicity of BT-A (a characteristic that has been reported particularly for INCO) [36,37,38,39].

No differences were found in goal achievement among patients with different characteristics (age, type of stroke, time since onset, or other variables), nor among those with varying types of pain or gait disturbances. So, all patients in this cohort benefit from botulinum toxin injection.

It is possible that some of the pain was due to degenerative osteoarticular conditions rather than spasticity alone; nevertheless, even in cases with mixed etiologies, patients experienced similar improvements. The benefits of BT-A in the pain of spastic patients may be related to reductions in muscular hyperactivity, inhibition of pain-related neurotransmitters (e.g., substance P, CGRP, and glutamate), reductions in peripheral and central sensitization, and anti-inflammatory effects. These mechanisms may help explain the observed improvements in pain outcomes [40,41,42,43,44].

These findings suggest that INCO and, probably, BT-A should be considered for pain management in patients with spasticity, regardless of the underlying cause.

It was interesting to find that in different populations, according to age, time from diagnosis, presence of pain, degree of spasticity, and mobility, a similar benefit was found with BT-A injection, without a statistical difference between groups.

None of the factors studied had a statistically significant impact on changes in the items Spasticity spectrum, Pain, Gait, Mobility, or ALDs. For this reason, we could not conclude that any factor had influenced the achievement of these good outcomes.

The study has some limitations. First, it is a retrospective analysis; it would be advisable to conduct a blinded randomized controlled trial with similar characteristics. The introduction of a control group would help exclude potential placebo effects and/or allow comparison of results between different BT-A formulations, while a blinded design would reduce possible assessor bias.

Although this is a comprehensive study (5-year follow-up, 20 variables), patients excluded due to missing information may have had different outcomes, as they may have experienced poorer results or difficulties accessing treatment. Similarly, although we included a heterogeneous population with different etiologies, this may have reduced the study-specific analytical power.

Regarding the outcome measures, the VAS is a subjective scale that does not differentiate between types of pain, and GAS depends on the assessor, making it difficult to replicate and susceptible to bias from the initial score definition. Nevertheless, both scores showed a clear trend toward improvement.

Finally, it would be important to conduct a multicenter study with different populations and analyses.

## 4. Conclusions

Pain and spasticity are very frequently observed problems in neurological conditions. These two conditions further impair the quality of life and functional abilities of patients who are already affected by weakness, imbalance, and other neurological deficits. Therefore, it is vital to control these symptoms to achieve the best possible outcome for each patient. Many treatments have been developed, and botulinum toxin type A is a class A evidence for focal spasticity. Meanwhile, studies for benefits in pain and with a long period of follow-up remain limited.

Our study shows that INCO can produce benefits of the same magnitude for patients with and without pain, and across different characteristics. Our good results encourage the regular use of INCO and BT-A in spastic patients with pain, irrespective of cause, as the magnitude of benefit appears consistent across different patient profiles.

This study is one of the studies with a longer period of follow-up and with a broad number of analyzed variables, which is very important for providing insights into the long-term effects of BT-A in these fields.

Future studies with control groups and randomized controlled trial designs are needed to confirm these results, and in other botulinum toxin formulations and pain subtypes.

## 5. Materials and Methods

To analyze the potential benefit of Incobotulinumtoxin A (Xeomin^®^, Merz Pharmaceuticals GmbH, Frankfurt, Germany) in pain, the spasticity spectrum, and functionality in spastic patients, a 5-year review of all clinical processes for all patients under these treatments between 2017 and 2022 was conducted.

Inclusion criteria were: adults (≥18 years), provision of written informed consent, treatment with Incobotulinumtoxin A for spasticity, functional impairment attributable to spasticity, clinical assessment during the 5-year study period, complete information for the 20 selected variables, and stable concomitant antispastic medication and rehabilitation program.

People who were treated with Incobotulinumtoxin A for other causes or with a lack of information were excluded.

Patients were treated every 6 months and evaluated at the time of injection and i month after each treatment.

We do not change the conservative rehabilitation treatments. So, people maintained their previous rehabilitation program with physiotherapy and/or occupational therapy and medication related to pain and spasticity.

A total of 20 variables were collected for the analysis: First treatment date, age, gender, etiology of spasticity, presence of spasticity in lower and upper limbs, number of affected limbs, spastic pattern in upper and lower limb, number of muscles treated in each appointment, number of different muscles treated in the total of sessions, number of treatments, dosage (minimum and maximum), presence of pain, visual analogue scale before and after treatments, Goal attainment scale (G.A.S.) before and after treatments, and Timed Up and Go Test before and after treatments.

Pain intensity level was evaluated by the visual analogue scale. It is a measurement tool that typically presents a 10 cm line with opposing descriptors at each end (e.g., “no pain”—0 to “worst pain imaginable”—10) and patients indicate their level of pain by marking a point on the line.

For subsequent analysis, pain was subcategorized into 3 levels: mild (<3 on VAS), moderate (4–6 on VAS), and severe (>7 on VAS).

Because patients have different neuromotor deficits and levels of function, we also follow up the benefits of treatment using the G.A.S. The main characteristic of this scale is that it allows the establishment of individualized criteria for success. We evaluate 3 realistic and specific goals for each patient, according to the patient/family principal’s desires. Each goal is then generally reevaluated on a 5-point scale (+2: much more than expected (Tscore = 70); +1: a little bit more than expected (Tscore = 60); 0: achieved as expected (Tscore = 50); −1: less than expected (Tscore = 40); −2: much less than expected (Tscore = 30). In our practice to have a more precise notion of benefits, we also catalogued specific ordinary values for each of 5 nominal degrees of the scale (see below; e.g., much more than expected (+2) for improvements above 50% the baseline).

Analysis of the Goal Attainment Scale was classified into 5 major groups: Pain (by VAS analysis), Spasticity spectrum (Spasticity, spams, or other involuntary movements), Mobility (change in active or passive range of motion), activities of daily living (ADLs), and Gait (speed by TUG).

For the interpretation of GAS in pain, spastic spectrum, and mobility, a value of 0 was attributed to a similar status or improvements of less than 25%. A value of +1 was attributed to improvements of 25% to 49%, and +2 to improvements greater than 50%. Values of −1 and −2 were attributed to worsening in the same magnitude (25 to 49% and >50%, respectively).

For the functional items, ADLs and Gait speed because it is a characteristic more difficult to achieve a different score was attributed. For ADLs, a value of 0 was given for a similar status or improvements in less than 2 ADLs. A value of +1 was used for the acquisition of 2 or more ADLS, and the value of +2 was used for more than 4. Values of −1 and −2 were attributed to worsening in the same magnitude.

For the item Gait, a value of 0 was given for a similar status or improvements of less than 15% on the TUG test. A value of +1 was attributed to improvements of 15–24% and +2 to improvements of greater than 25%. Values of −1 and −2 were attributed to worsening in the same magnitude (15–24% and >25%, respectively).

The sociodemographic data and other baseline characteristics of the study are described using descriptive statistics. Continuous variables are described by measures of central tendency (mean and median) and by measures of dispersion (standard deviation, variance, and interquartile range), while categorical variables are described by absolute and relative frequencies.

The normality of the results was assessed using the Shapiro–Wilk and Kolmogorov–Smirnov tests.

To analyze the possible factors influencing changes in the sub-items (pain, functionality in GAS, spasticity spectrum, mobility, and Gait), a univariate logistic regression model was first performed, considering sociodemographic characteristics and related aspects as possible predictors. Variables with a significance level of *p* < 0.10 in the univariate analysis were then entered into a multivariate logistic regression model using a backward stepwise selection procedure. Statistical significance was set at *p* < 0.05.

This study was approved by the Hospital Ethical Committee and reported in accordance with the STROBE guidelines.

After the collected data in Excel, a random number was assigned to each patient to protect confidentiality. All data were then passed to SAS 9.4 and reevaluated by an independent statistical committee.

## Figures and Tables

**Figure 3:**
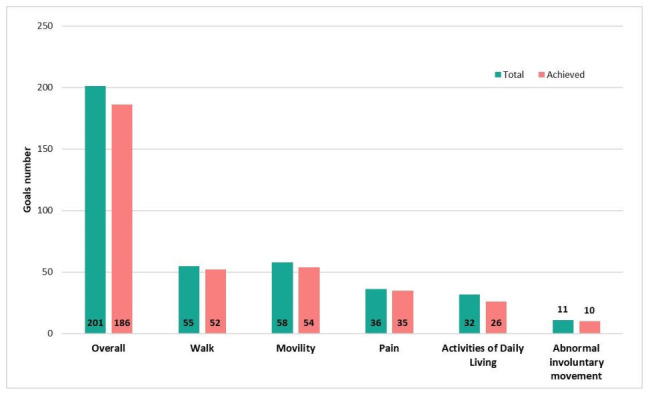
Patient goals and achievements.

**Figure 4:**
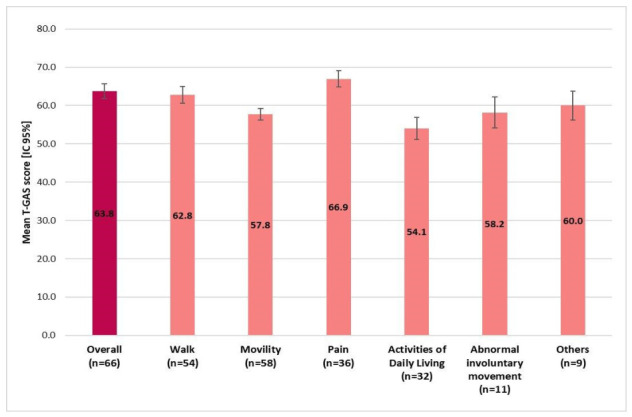
Accumulated Mean T-score GAS in different areas.

**Figure 5:**
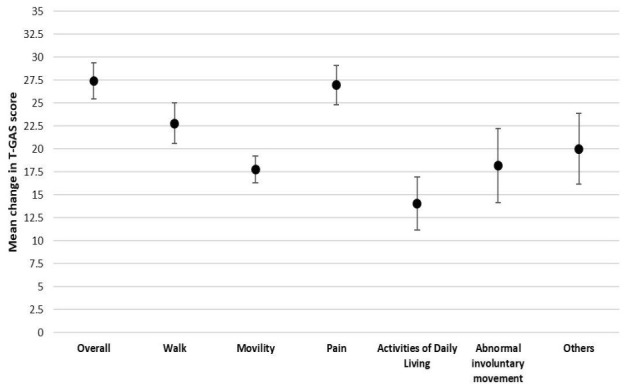
Mean T-score GAS change in different areas.

**Table 1:** Etiology of spasticity.

Underlying Diseases	Total N = 67
Stroke	40 (59.7%)
Cerebral palsy	9 (13.4%)
Cervical spondylotic myelopathy/Spinal cord lesion	9 (13.4%)
Multiple sclerosis	3 (4.5%)
Traumatic brain injury	2 (3.0%)
Hereditary spastic paraplegia	2 (3.0%)
Other *	2 (3.0%)

* Other diagnoses included encephalitis and nervous system infection.

**Table 2:** Injected Muscle Groups.

Treated Muscle Groups	Total N = 67
**Upper limb muscle groups treated**	**45 (67.2%)**
Wrist flexors	36 (80.0%)
Finger flexors	34 (75.6%)
Elbow flexors	26 (57.8%)
Shoulder adductors	23 (51.1%)
Forearm pronators	20 (44.4%)
Elbow extensors	7 (15.6%)
Internal rotators of the shoulder	6 (13.3%)
Shoulder extensors	2 (4.4%)
Shoulder flexors	1 (2.2%)
Forearm supinators	1 (2.2%)
**Lower limb muscle groups treated**	**59 (88.1%)**
Ankle plantar flexors	56 (94.9%)
Knee extensors	26 (44.1%)
Toe flexors	15 (25.4%)
Hip adductors	12 (20.3%)
Ankle invertors	10 (16.9%)
Hallux extensors	8 (13.6%)
Knee flexors	5 (6.5%)
Internal rotators of the hip	4 (6.8%)
Hip flexors	1 (1.7%)
Ankle invertors	1 (1.7%)

## Data Availability

Data supporting reported results can be found in this link: https://drive.google.com/drive/folders/1BuJ6wbZvH504hRYe00NPnfzJpdyn0bP9?usp=drive_link (accessed on 1 December 2024).

## Abbreviations

ADLs	Activities of Daily Living
BT-A	Botulinum toxin type A
CGRP	Calcitonin Gene-Related Peptide
GAS	Goal Attainment Scaling
E.G.	For example
IASP	International Association for the Study of Pain
INCO	Incobotulinumtoxin A
SCI	Spinal Cord injury
TUG	Timed Up and Go Test
VAS	Visual Analogue Scale
